# Efficacy of physical therapy interventions on quality of life and upper quadrant pain severity in women with post-mastectomy pain syndrome: a systematic review and meta-analysis

**DOI:** 10.1007/s11136-021-02926-x

**Published:** 2021-06-29

**Authors:** Priya Kannan, Hiu Ying Lam, Tsz Kiu Ma, Chiu Ngai Lo, Ting Yan Mui, Wing Yan Tang

**Affiliations:** Department of Rehabilitation Sciences, The Hong Kong Polytechnic University, Hung Hom, Kowloon, Hong Kong

**Keywords:** Acupuncture, Decongestive therapy, Exercise, Post-mastectomy pain syndrome, Quality of Life

## Abstract

**Purpose:**

To determine the efficacy of physical therapy interventions on quality of life (QoL) and pain severity in post-mastectomy pain syndrome (PMPS).

**Methods:**

Multiple databases were searched from database inception to October 2020. Searches were limited to human studies published in either English or Chinese in peer-reviewed journals with full text available for randomized controlled trials conducted on females. Trials comparing the effectiveness of physical therapy interventions against control conditions on QoL and pain were included.

**Results:**

Eighteen trials were included in the review. The pooled analysis of the four exercise trials revealed a significant effect of the intervention on general [standardized mean difference [SMD]: 0.87 (95%CI: 0.36, 1.37); *p* = 0.001], physical [SMD: 0.34 (95%CI: 0.01, 0.66); *p* = 0.044], and mental health components [SMD: 0.27 (95%CI: 0.03, 0.51); *p* = 0.027] of QoL compared with the control condition. Meta-analyses of six exercise trials, two myofascial release trials, and two acupuncture trials revealed a significant improvement in pain severity in the treatment group than in the control group. However, meta-analyses of two studies revealed a non-significant effect of compression therapy compared to control on pain severity.

**Conclusion:**

Our meta-analyses found that exercise is beneficial for improving the QoL and pain severity of women with PMPS. Future studies are needed to determine the optimal parameters for exercise interventions designed to improve QoL and pain severity in women with PMPS. The effect of acupuncture, myofascial release, and compression therapy remains inconclusive, and future research is required to validate the effect of these interventions on PMPS.

**Supplementary Information:**

The online version contains supplementary material available at 10.1007/s11136-021-02926-x.

## Introduction

Post-mastectomy pain syndrome (PMPS) is defined as chronic neuropathic pain affecting the upper quadrant (including the breast, chest wall, axilla, and ipsilateral medial arm) after breast cancer surgery [1]. PMPS affects 20%–68% of post-mastectomy patients worldwide [2]. PMPS occurs following all kinds of breast surgery, including mastectomy, lumpectomy, and quadrantectomy with axillary lymphadenectomy [3], and persist for at least six months post-operatively [4]. It is associated with damage to nervous tissue, in particular the intercostobrachial, medial pectoral, lateral pectoral, thoracodorsal, or long thoracic nerves [5].

Post-mastectomy pain has been reported to have adverse impacts on quality of life (QoL), including impaired physical functioning and increased psychosocial distress [6]. Surgery-related arm symptoms (such as lymphedema, pain, numbness, stiffness, and limited shoulder range of motion) can cause functional impairment, lowering the QoL of women with PMPS [7]. In addition, the occurrence of pain in cancer survivors represents a continuous memory of both the disease and the treatment and can be viewed by some survivors as a sign of residual disease, leading to fears of worsening or recurrence [8]. Even in the absence of disease progression, these factors contribute to substantial psychophysical distress among cancer survivors who experience pain, with negative effects on QoL [8]. The efficacy of conservative therapies on QoL among women who underwent breast cancer treatment [9] or who experienced lymphedema following breast cancer therapy [10] was previously evaluated in meta-analytic reviews. However, previous meta-analytic reviews either evaluated the efficacy of single interventions, did not evaluate the QoL of women with PMPS, or were published more than five years ago [9, 10]. The current meta-analytic review is the first to evaluate QoL among women with PMPS and include all physical therapy interventions.

Physical therapy interventions that have been proposed for reducing post-mastectomy pain include exercise, acupuncture, cryotherapy, biofeedback, transcutaneous electrical nerve stimulation, and massage therapy [11]. A systematic review investigating the effectiveness of post-operative physical therapy interventions on upper limb pain in breast cancer patients identified exercise (i.e., manual stretching and active exercises) as effective for treating post-operative breast cancer pain [12]. However, the review did not include a meta-analysis and was published over five years ago [12]. The objective of this meta-analytic review was to determine the efficacy of physical therapy interventions on both QoL and upper quadrant pain in women with PMPS.

## Materials and methods

This meta-analytic review was developed and is reported in accordance with the Preferred Reporting Items for Systematic Reviews and Meta-Analyses (PRISMA) guidelines [13]. This meta-analytic review is registered in the PROSPERO registry (CRD42020179900).

### Search strategy and study screening

Databases—including AMED, CINAHL, Cochrane Central Register of Controlled Trials, EMBASE, Medline, PEDro, PubMed, Scopus, and Web of Science were searched from database inception until April 2020. Searches were updated in October 2020. We utilized three comprehensive search themes: breast cancer; physical therapy interventions; and randomized controlled trial [RCT] to retrieve potentially relevant articles. Searches were limited to (1) human studies; (2) RCTs conducted on females; and (3) full-text studies published in either English or Chinese (traditional or simplified) in scholarly peer-reviewed journals. The specific search strategy for the Medline database is presented in supplementary Appendix 1. The reference lists of relevant systematic reviews were also manually searched in order to identify any other potentially eligible trials. Disagreements regarding study selection were resolved by discussion between the two reviewers. A third reviewer was consulted for any unresolved disagreements.

The inclusion criteria were: (1) RCTs (parallel, crossover, or pilot) that compared the effect of various physical therapy interventions to control (no treatment, standard care, sham, placebo, usual care, or active control) on QoL and upper quadrant pain in women with PMPS and; (2) trials that utilized either the 36-Item Short Form Survey (SF-36) or 12-Item Short Form Survey (SF-12) for measurement of QoL or the Visual Analog Scale (VAS), Numeric Pain Rating Scale (NPRS), or Brief Pain Inventory (BPI) (short form, Q3-6) for measurement of pain severity. A piloting of the study selection process (inclusion and exclusion criteria) was performed prior to commencing this meta-analytic review. A quick piloting process is recommended to enable themes and determine which standardized outcomes have been included in a sample of studies that examine the intervention of interest [14]. The measures of QoL and pain that were utilized in the current meta-analytic review were chosen based on the results of the piloting process. Studies were excluded if they (1) compared two physical therapy interventions or two different treatment parameters, (2) combined more than one intervention in either treatment group or control group, and (3) involved subjects with other cancers (such as ovarian, uterine, etc.) in addition to breast cancer. Trials were not excluded based on the year of publication.

### Data extraction

Data extraction was conducted by two independent reviewers. Relevant data extracted from each study included the following: last name of the first author, publication year, country, mean age of participants, sample size in each study group, intervention and control, outcome measures, and data [mean and standard deviation (SD)] reported at baseline (pre-intervention) and at the end of the longest follow-up period. If any study reported results as non-parametric data (i.e., median and interquartile range) unsuitable for meta-analysis, Bland's and Wan’s methods were applied to calculate the mean and SD [15, 16].

### Quality assessment

Trials were not excluded on the basis of quality, although quality was taken into consideration when interpreting the results. The methodological quality of the RCTs was evaluated by two independent reviewers using the Physiotherapy Evidence Database (PEDro) scoring system. Trials that scored ≥ 6 were considered to be of high quality, scores of 4–5 were considered fair quality, and scores < 4 were considered poor quality [17].

The quality of the evidence in each RCT was assessed using the Grading of Recommendations, Assessment, Development, and Evaluation (GRADE) tool. GRADE profiler software (version 3.6.1, http://tech.cochrane.org/revman/other-resources/gradepro/download) developed by the GRADE group was used to rate the quality of evidence. Five factors were considered for rating the quality of evidence: risk of bias, imprecision, inconsistency, indirectness, and publication bias [18]. Trials were downgraded for risk of bias for the following reasons: lack of allocation concealment, lack of assessor/therapist blinding, loss of > 15% of participants over follow-up, selective outcome reporting, cessation of the study for a benefit, and failure to perform an intention-to-treat analysis [19]. Trials were downgraded for precision level if there was minimal or no overlap of confidence intervals (CIs) or if the total number of participants included in the review was smaller than the sample size required for an adequately powered trial [20]. Optimal Information Size (OIS) was used to determine the necessary sample size required for an adequately powered trial. To inform this decision, the OIS for a two-arm parallel-group trial was calculated using data from a previous study, assuming an α of 0.05 and 80% power (β = 0.2) [21]. Trials were downgraded for inconsistency if there was wide variations in point estimates across studies, wide CIs or evidence of statistical heterogeneity as indicated by a large *I*^2^ value (> 50%) [22]. Trials were downgraded for indirectness if there was a difference between the populations, interventions, or outcome measures (surrogate outcomes) across trials [23]. Trials were downgraded for publication bias if they were commercially funded, likely to be sponsored by industry, or if the authors shared a conflict of interest [24].

### Statistical analysis

Meta-analyses were conducted using Comprehensive Meta-Analysis software, version 3. Trials of similar interventions and outcome measures were pooled together. For QoL, the differences in mean and 95% CI were calculated. For pain measurement, Hedges’ g [standardized mean difference (SMD)] and 95% CIs were computed because of different measurement scales across trials. Statistical heterogeneity was assessed using the Chi-square test (*I*^2^). A *p* value ≤ 0.05 was defined as statistical significance.

## Results

**Fig. 1:**
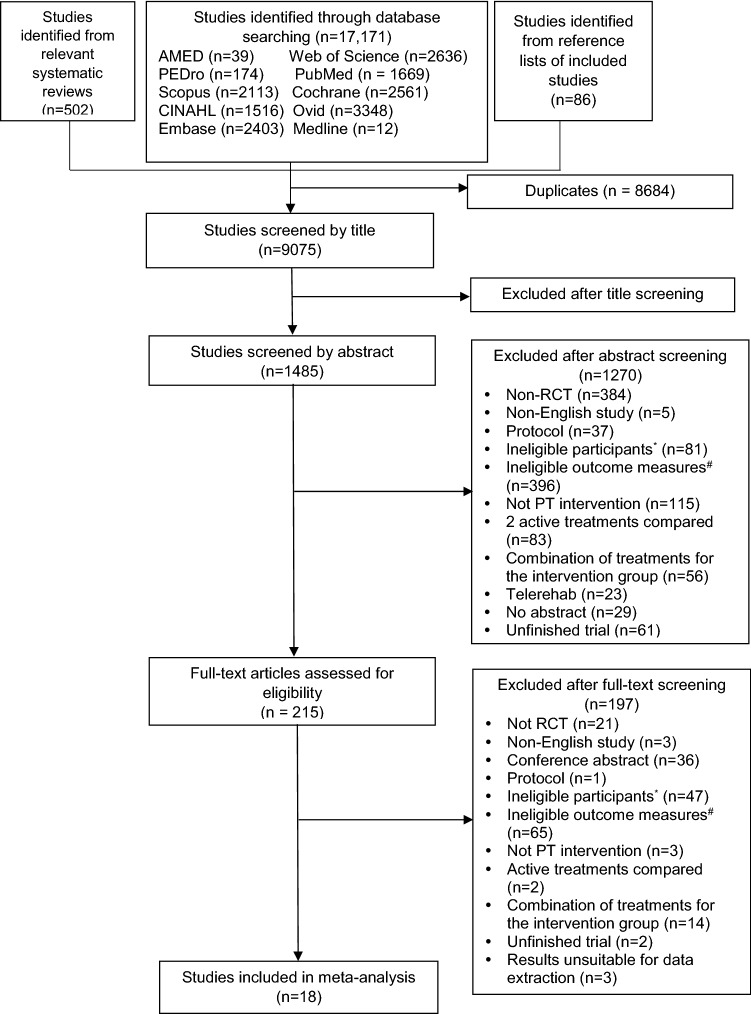
Flowchart of the study selection. *PT* Physical therapy, *Ineligible women: Women without upper quadrant pain, #Ineligible outcome measures: Outcome measures out of scope of interest of the current meta-analytic review

Figure 1 summarizes the study selection process based on the PRISMA approach. Trials excluded at the full-text screening stage and the reasons for exclusion are listed in supplementary Appendix 2. Electronic and manual searching identified 17,759 articles. Eighteen trials met the inclusion criteria and were included in the meta-analytic review.

### Characteristics of included trials

The characteristics of the included trials are summarized in Table 1. Data from 1098 women were collected across the 18 trials included in the meta-analysis. The sample size of included trials ranged from 23 to 291. Mean participant age ranged from 45.6 to 67.8 years. Interventions in the included studies are exercise (*n* = 10), myofascial release (*n* = 2), acupuncture (*n* = 2), compression therapy (*n* = 2), self-administered complex decongestive therapy (CDT; *n* = 1), and neuromuscular taping (NMT; *n* = 1). Of the 18 included trials, four reported QoL and 14 reported pain severity.

**Table 1:** Characteristics of included trials (*n* = 18)

First author, year, country of study	Mean age of participants (SD); sample size of each group	Intervention	Control	Outcome measure(s)	Results (time points of assessment): mean (SD)
Ammitzbøll [29], 2019,.Denmark	Exp: 53 (10) Con: 52 (10) Exp: *n* = 82 Con: *n* = 76	Progressive resistance training exercise program - Biceps, shoulder abductor & extensors, triceps, lower limb & core - Weeks 1–20: supervised by physiotherapist - Weeks 30–50: self-administered exercise - 3 Times/week × 50 weeks Intensity: - Weeks 1–4: 25RM × 20 repetitions × 2 sets - Weeks 5–20: gradual progression - Weeks 21–50: 10RM × 10–12 repetitions × 3sets	Post op usual care Mobility exercise and manual therapy	Pain severity: NPRS	Pre Exp: 2.90 (0.33) Con: 2.90 (0.33) Post (12 months) Exp: 1.34 (0.43) Con: 1.90 (0.45)
Andersen-Hammond [30],.2020, Canada	Exp: 56.3 (9.9) Con: 53.0 (10.3) Exp: *n* = 22 Con: *n* = 26	Nerve gliding exercise - 5–10 min × 3 times daily Stretching and ROM exercise - Neck & UL and axillary webbing exercise Education - Symptoms management, safety and protection	Usual care	Pain severity: NPRS	Pre* Exp: 1.13 (1.11) Con: 1.45 (1.80) Post (6 months)* Exp: 0.38 (0.41) Con: 0.31 (0.35)
Cantarero-Villanueva [31], 2012, Spain	Exp: 48 (8) Con: 47 (9) Exp: *n* = 33 Con: *n* = 33	Water exercise program - 1 h (10 min warm-up; 35 min aerobic, low intensity endurance core stability and strength training; 15 min cool down) - Warm pool (28–31 °C) with water depth 1.4–1.8 m - 3 times/week × 8 weeks	Usual care Recommendations related to nutrition, lifestyle behaviors, and exercise	Pain severity: VAS (0–100)	*Neck pain* Pre Exp: 40 (31) Con: 39 (21) Post (8 weeks) Exp: 12 (15) Con: 42 (23) *Shoulder/ axillary pain* Pre Exp: 27 (33) Con: 38 (35) Post (8 weeks) Exp: 12 (13) Con: 43 (33)
Castro-Martín [35], 2017, Spain	50.14 (8.81) Exp: *n* = 21 Con: *n* = 21	Myofascial induction (fascial unwinding) on upper limb - 30 min/session × 1 session - Washout period between myofascial induction and placebo: 4 weeks	Unplugged pulsed shortwave therapy - 30 min/ session × 1 sessions	Pain severity: VAS	*Cervical* Pre Exp: 5.62 (2.54) Con: 4.67 (3.02) Post (immediately after treatment) Exp: 3.71 (2.51) Con: 3.33 (2.67) *Affected arm* Pre Exp: 4.90 (2.62) Con: 3.95 (2.01) Post (immediately after treatment) Exp: 2.62 (2.42) Con: 2.95 (2.22)
Conejo [40], 2018, Spain and Australia	Exp: 67.8 ~ Control: 64.8 ~ Exp: *n* = 20 Con: *n* = 20	Neuromuscular taping (NMT) - Area of pain: cervical, lumbosacral, wrist forearm, or both - 3 Sessions: beginning of intervention, reapply at day 7 and week 5 Decalogue of health advice	Sham NMT in painful areas Decalogue of health advice	Pain severity: VAS	Pre Exp: 7.40 ~ Control: 6.65 ~ Post (5 weeks) Exp: 4.90 ~ Control: 6.45 ~
Dong [21], 2019, China	Exp: 48.0 (5.5) Con: 51.6 (7.5) Exp: *n* = 26 Con: *n* = 24	Muscle training - 30 min (5 min warm-up; 20 min muscle training; 5 min cool down) - 3 times/week × 12 weeks - 1st month: endurance - 2nd month: strength - 3rd month: muscle function Cardio-pneumatic endurance training - 4 times/week × 12 weeks Post-operative rehabilitation knowledge	Traditional treatment and rehabilitation Recommendations from the National Institute for Health and Care Excellence (NICE) clinical guidance	QoL: SF-36	*SF-36: general* Pre Exp: 65.96 (15.85) Con: 57.21 (19.80) Post (12 weeks) Exp: 73.38 (18.16) Con: 63.08 (18.90) *SF-36: physical* Pre Exp: 32.69 (38.58) Con: 59.38 (42.23) Post (12 weeks) Exp: 57.69 (37.93) Con: 53.13 (41.25) *SF-36: mental* Pre Exp: 51.08 (6.23) Con: 51.83 (6.62) Post (12 weeks) Exp: 54.62 (4.92) Con: 49.83 (5.53)
García-Soidán [26], 2020, Spain	Exp 1: 63 (7) Exp 2: 62 (2) Exp 3: 64 (7) Con: 65 (4.6) Exp 1: *n* = 74 Exp 2: *n* = 65 Exp 3: *n* = 79 Con: *n* = 73	55–60 min × 2 sessions/week × 2 years Exp 1: Strength training group - 10 min warm-up; 30–40 min resistance exercise; 10 min stretching - 8 resistance exercises for lower and upper limb large muscle group Intensity: - Weeks 1–6: 50–60% 1RM × 12 repetitions × 2 sets - Weeks 7–8: 60% 1RM × 20 repetitions × 2 sets - Weeks 100–104: 60–80% 1RM × 10 repetitions × 3 sets Exp 2: Aqua fitness group - 5 min warm-up; 25 min aerobic exercise; 10 min resistance exercise; 10 min game; 5 min stretching - Pool depth: 1.4–1.75 m - Resistance exercise of chest, shoulder & dorsal region, arm & forearm, lower limbs & abdominal muscles Intensity: - Weeks 1–2: low intensity - Weeks 3–12: progressive increase Exp 3: Aerobic exercise group - 10 min warm-up; 40 min choreographed aerobic exercise; 5 min stretching - Strengthening exercise of upper and lower limbs large muscle groups without loads: 12 repetitions × 2 sets	Control group No change in lifestyle and no new physical activity incorporated	QoL: SF-12	*SF-12: general* Pre Exp 1: 41.8 (9.3) Exp 2: 38.1 (8.3) Exp 3: 40.3 (9.9) Con: 42.3 (9.3) Post (2 years) Exp 1: 44.4 (13.7) Exp 2: 39.8 (13.7) Exp 3: 43 (11.5) Con: 25.6 (15.2) *SF-12: physical* Pre Exp 1: 45.6 (4.2) Exp 2: 45.1 (4.1) Exp 3: 44.8 (3.8) Con: 43.8 (4.5) Post (2 years) Exp 1: 47.5 (7.8) Exp 2: 47.8 (7) Exp 3: 47.3 (8.5) Con: 46.9 (7.4) *SF-12: mental* Pre Exp 1: 38.4 (1.4) Exp 2: 38.9 (4.2) Exp 3: 39 (4.5) Con: 38.1 (5.4) Post (2 years) Exp 1: 44 (4.5) Exp 2: 43.5 (4.1) Exp 3: 43.2 (3.8) Con: 42.2 (4.5)
Hansdorfer-Korzon [41], 2016, Poland	Exp: 62.4 (12.9) Con: 62.5 (12.0) Exp: *n* = 19 Con: *n* = 18	Low-pressure compression corsets - 7 months	No physiotherapy treatment	Pain severity: VAS	Pre^ Exp: 0/19 Con: 0/18 Post (7 months) Exp: 11/19 Con: 6/18
Hwang [32], 2008, Korea	Exp: 46.3 (7.5) Con: 46.3 (9.5) Exp: *n* = 17 Con: *n* = 20	Supervised exercise programme - 50 min (10 min warm-up; 30 min shoulder stretching, aerobic & resistance exercise; 10 min cool down) - 3 times/week × 5 weeks Intensity: - Moderate: 50–70% HR maximum	Self-shoulder stretching exercise and encouraged to continue with normal activities	Pain severity: VAS	Pre Exp: 35.0 (3.9) Con: 26.5 (4.5) Post (5 weeks) Exp: 24.3 (5.3) Con: 29.6 (4.9)
Irwin [27], 2008, USA	Exp: 56.5 (9.5) Con: 55.1 (7.7) Exp: *n* = 37 Con: *n* = 37	Supervised aerobic exercise training program - 60–80% HR maximum - 30 min × 3 times/week × 6 months Home-based aerobic training program - 30 min × 2 times/week × 6 months	Usual care Without study’s physical activity program	QoL: SF-36	*SF-36: physical* Pre Exp: 50.2 (6.6) Con: 48.0 (7.5) Post (6 months) Exp: 50.0 (6.4) Con: 48.0 (7.6) *SF-36: mental* Pre Exp: 49.8 (8.4) Con: 48.2 (11.1) Post (6 months) Exp: 50.6 (10.9) Con: 47.4 (12.0) *SF-36: general* Pre Exp: 49.8 (7.2) Con: 51.5 (8.0) Post (6 months) Exp: 50.0 (8.8) Con: 51.7 (8.4)
Johansson [42], 2020, Sweden	Exp: 61.9 (7.6) Con: 61.3 (9.6) Exp: *n* = 14 Con: *n* = 9	Compression therapy - Sports bra of compression type with firm pressure flattening the breast - Worn during daytime but not at night - 9 months	Ordinary bras used during daytime Allowed to use loose-fitted sports bras	Pain severity: VAS (0–100)	Pre^#^ Exp: 2 (4.12) Con: 16.67 (28.00) Post (9 months) Exp: 10.67 (12.36) Con: 24.67 (47.23)
Lee [33], 2010, Korea	Exp 1: 47.5 (5.1) Exp 2: 45.6 (7.0) Con: 47.6 (9.2) Exp 1: *n* = 13 Exp 2: *n* = 13 Con: *n* = 18	90 min (5 min warm-up; 40 min stretching; 40 min strengthening; 5 min cool down) 1 time/week × 8 weeks Exp 1: Scapula-oriented shoulder exercise group - Shoulder ROM exercise, stretching of neck muscle and pectoralis - Elastic band strengthening exercise of scapular and shoulder muscle - Ball exercise for shoulder stabilization Exp 2: General exercise group - Stretching exercise of neck, shoulder, trunk - Strengthening exercise of shoulder & core muscle	Historical control group A leaflet guiding self-care was provided	Pain severity: VAS, BPI	*VAS (rest; active)* Pre Exp 1: 0.5 (0.8); 2.3 (1.2) Exp 2: 1.0 (1.4); 3.0 (2.4) Con: 1.4 (2.0); 2.4 (2.0) Post (8 weeks) Exp 1: 0.5 (0.9); 1.8 (1.7) Exp 2: 0.2 (0.6); 1.7 (1.8) Con: 1.2 (1.5); 2.5 (1.5) *BPI* Pre Exp 1: 1.8 (1.2) Exp 2: 2.3 (1.9) Con: 2.2 (2.0) Post (8 weeks) Exp 1: 1.2 (1.3) Exp 2: 1.3 (1.5) Con: 1.9 (1.1)
Ligabue [39], 2019, Italy	Exp: 56. 8 (8.8) Con: 57.1 (9.8) Exp: *n* = 20 Con: *n* = 21	Self-administered complex decongestive therapy - Manual lymphatic self-drainage - Self-bandage - Breathing exercises - Mobilization exercises - Muscle reinforcement exercises - Muscle contracture management - Education about the changes that occur post-lymphedema - 10 sessions × 4 weeks	Usual care Discussion and briefing of leaflet regarding exercises, behavioral and hygienic standards	Pain severity: NPRS	Pre Exp: 4.3 (2.6) Con: 3.8 (2.8) Post (6 months) Exp: 2.1 (2.5) Con: 3.8 (3.3)
Lu [38], 2020, USA	Exp: 54.0 ~ Con: 53.5 ~ Exp: *n* = 14 Con: *n* = 17	Acupuncture - Needle size and length: 0.20 × 25 mm & 0.25 × 40 mm - 30 min × 18 sessions × 8 weeks Week 1: manual acupuncture - Acupoints: bilateral SP9, ST36, K3, LI11, Sp6, LR3, second Baxie, TW5, Yin Tang (depends on participants’ tolerance) Week 2–8: electro acupuncture - Acupoints: bilateral TW5, second Baxie and/ or SP6, LR - Alternating frequency: 2-10 Hz	Wait list control group Received no acupuncture treatment in the first 8 weeks	Pain severity: BPI-SF	Pre Exp: 3.9 (1.6) Con: 3.7 (2.0) Changes (8 weeks) Exp: -1.1 (1.7) Con: 0.3 (1.5)
Nyrop [34], 2017, USA	Exp: 63.3 (6.9) Con: 64.4 (9.7) Exp: *n* = 24 Con: *n* = 29	Walk With Ease-Breast Cancer - Walk on their own or with others at safe pace - 150 min/week × 6 weeks - Workbook and brochure with strategies	Wait list control	Pain severity: VAS	Pre Exp: 5.22 (2.43) Con: 4.95 (2.43) Post (6 weeks) Exp: 4.47 (2.53) Con: 4.82 (2.44)
Paulo [28], 2019, Brazil	Exp: 63.2 (7.1) Con: 66.6 (9.6) Exp: *n* = 18 Con: *n* = 18	Exercise program 1. Aerobic treadmill exercise - 30 min Intensity: - Week 1–8: 60–65% HR maximum - Weeks 9–20: 65–70% HR maximum - Weeks 21–30: 70–75% HR maximum - Weeks 31–36: 75–80% HR maximum 1. Resistance exercise - 40 min - 3 times/week × 9 months Intensity: - Momentary exhaustion Health education lecture - 90 min - 1 time/month × 9 months	Stretching and relaxation exercises 10-15 s each 45 min 2 times/week × 9 months	QoL: SF-36	*SF-36: general* Pre Exp: 84.9 (10.8) Con: 83.8 (9.2) Post (9 months) Exp: 96.4 (4.7) Con: 87.3 (10.3) *SF-36: physical* Pre Exp: 75.8 (13.4) Con: 73.9 (11.5) Post (9 months) Exp: 93.9 (8.8) Con: 75.2 (12.6) *SF-36: mental* Pre Exp: 84.6 (8.5) Con: 79.9 (8.6) Post (9 months) Exp: 85.6 (13.3) Con: 77.3 (8.4)
Quinlan-Woodward [25], 2016, USA	Exp: 53.7 (9.4) Con: 62.5 (11.5) Exp: *n* = 10 Con:* n* = 14	Post op acupuncture - Acupoints: based on presenting symptoms - Average needling time: 36 min - At most 2 times during post op hospitalization within ≥ 12 h apart	Usual care	Pain: NPRS	Pre Exp: 4.2 (1.01) Con: 3.67 (2.13) Post (time-point of assessment NR) Exp: 1.6 (1.35) Con: 2.64 (2.31)
Serra-Añó [36], 2018, Spain	Exp: 53.15 (10.91) Con: 54.36 (6.86) Exp: *n* = 11 Con: *n* = 13	Myofascial release - 3-dimensional fascial movement with light pressure and stretching of connective tissue - 4 maneuver: sterno-pectoral, global pectoral, pectoral, subscapularis - 10 min/technique - 50 min/session × 1 session/week × 4 weeks	Placebo manual lymphatic drainage Gentle, superficial manipulation of axillary lymph nodes in chest and arm	Pain: VAS	Pre Exp: 6.48 (1.52) Con: 4.95 (2.09) Post (2 months) Exp: 3.62 (3.07) Con: 4.68 (1.61)

*BPI* Brief pain inventory, *Con* Control group, *Exp* Experimental group, *NPRS* Numerical Pain Rating Scale, *QoL SF-12* Quality of life 12-Item short form questionnaire, *QoL SF-36* Quality of life 36-Item short form questionnaire, *ROM* range of motion, *RM* repetition maximum, *UL* upper limb, *VAS* visual analog scale

*SD and mean calculated were from median, interquartile range, minimum, maximum, and sample size using Bland’s method

^^^The study only reported the number of patients with and without reduction in pain; the fraction shows the number of patients with pain reduction in VAS)/(total number of patient in the sub-group)

^#^SD and mean were calculated from median, interquartile range, and sample size using Wan’s method

^~^Standard deviation is not reported

### Methodological quality

The PEDro quality of the included trials is presented in Table 2. The mean PEDro score of the 18 trials was 6.2 out of 10. Of the 18 included trials, 12 were of high methodological quality, five of fair quality and one trial was of poor quality. Among the 18 included trials, 17 lacked therapist blinding, nine did not report allocation concealment, nine lacked intention-to-treat analysis, eight lacked assessor blinding, and six trials lost > 15% of participants to follow-up.

**Table 2:** PEDro scores of included trials (*n* = 18)

Trial	Random allocation	Concealed allocation	Baseline comparability	Participant blinding	Therapist blinding	Assessor blinding	Adequate follow-up	Intention-to-treat analysis	Between-group comparisons	Point estimate and variability	Total score (0–10)
Ammitzbøll [29], 2019	Y	Y	Y	N	N	Y	Y	Y	Y	Y	8
Andersen-Hammond [30], 2020	Y	Y	Y	N	N	Y	N	N	Y	Y	6
Cantarero-Villanueva [31], 2012	Y	Y	Y	N	N	Y	Y	Y	Y	Y	8
Castro-Martín [35], 2017	Y	N	Y	N	N	N	Y	N	Y	Y	5
Conejo [40], 2018	Y	Y	Y	N	N	N	Y	Y	Y	Y	7
Dong [21], 2019	Y	Y	Y	N	N	Y	N	N	Y	Y	6
García-Soidán [26], 2020	Y	Y	Y	N	N	Y	Y	N	Y	Y	7
Hansdorfer-Korzon [41], 2016	Y	N	N	N	N	N	N	N	Y	Y	3
Hwang [32], 2008	Y	N	Y	N	N	N	Y	N	Y	Y	5
Irwin [27], 2008	Y	Y	Y	N	N	Y	Y	Y	Y	Y	8
Johansson [42], 2020	Y	N	Y	N	N	Y	Y	N	Y	Y	6
Lee [33], 2010	Y	N	Y	N	Y	Y	N	N	Y	Y	6
Ligabue [39], 2019	Y	Y	Y	N	N	Y	Y	Y	Y	Y	8
Lu [38], 2020	Y	N	Y	N	N	N	N	Y	Y	Y	5
Nyrop [34], 2017	Y	N	Y	N	N	N	Y	Y	Y	Y	6
Paulo [28], 2019	Y	N	Y	N	N	N	N	Y	Y	Y	5
Quinlan-Woodward [25], 2016	Y	N	Y	N	N	N	Y	N	Y	Y	5
Serra-Añó [36], 2018	Y	Y	Y	N	N	Y	Y	Y	Y	Y	8

*Y* Yes, *N* No

### Quality of evidence (GRADE)

The GRADE evidence profiles for individual interventions on each outcome measure are shown in Table 3. Serious risk of bias was the main factor contributing to downgraded quality of evidence, and this was identified by both the GRADE assessment and the PEDro scale.

**Table 3:** GRADE evidence profile and summary of findings (SoF)

Quality assessment							No of patients		Effect		Quality
No of studies	Design	Risk of bias	Inconsistency	Indirectness	Imprecision	Publication Bias	Intervention	Control	Relative(95% CI)	Absolute	
Exercise vs. control—outcome: quality of life (general health) (measured with: SF-36, SF-12; better indicated by higher values)											
4 [21, 26–28]	randomized trials	very serious^i^	very serious^j^	no serious indirectness	no serious imprecision	undetected	299	152	–	SMD's g 0.865 higher (0.360 to 1.371 higher)	⊕ ⊝ ⊝ ⊝ VERY LOW ^i, j^

Exercise vs. control—outcome: quality of life (physical health) (measured with: SF-36, SF-12; better indicated by higher values)											
4 [21, 26–28]	randomized trials	very serious^i^	very serious^k^	no serious indirectness	no serious imprecision	undetected	299	152	–	SMD's g 0.335 higher (0.009 to 0.661 higher)	⊕ ⊝ ⊝ ⊝ VERY LOW ^i, k^

Exercise vs. control—outcome: quality of life (mental health) (measured with: SF-36, SF-12; better indicated by higher values)											
4 [21, 26–28]	randomized trials	very serious^i^	very serious^l^	no serious indirectness	no serious imprecision	undetected	299	152	-	SMD's g 0.270 higher (0.030 to 0.510 higher)	⊕ ⊝ ⊝ ⊝ VERY LOW ^i, l^

Compression therapy vs. control—outcome: pain (measured with: VAS; better indicated by lower values)											
2 [41, 42]	randomized trials	very serious^f^	no serious inconsistency	no serious indirectness	no serious imprecision	undetected	33	27	–	SMD's g 0.292 higher (0.260 lower to 0.845 higher)	⊕ ⊕ ⊝ ⊝ LOW ^f^

NMT vs. sham-NMT—outcome: pain (measured with: VAS; better indicated by lower values)											
1 [32]	randomized trials	serious^g^	no serious inconsistency	no serious indirectness	no serious imprecision	undetected	20	20	–	SMD 2.812 lower (3.686 to 1.938 lower)	⊕ ⊕ ⊕ ⊝ MODERATE ^g^

CDT vs. control—outcome: pain (measured with: NPRS; better indicated by lower values)											
1 [39]	randomized trials	serious^h^	no serious inconsistency	no serious indirectness	no serious imprecision	undetected	20	21	–	SMD 0.749 lower (1.382 to 0.115 lower)	⊕ ⊕ ⊕ ⊝ MODERATE ^h^

Myofascial release vs. placebo—outcome: pain (measured with: VAS; better indicated by lower values)											
2 [35, 36]	randomized trials	serious^a^	no serious inconsistency	no serious indirectness	no serious imprecision	undetected	32	34	–	SMD's g 0.649 lower (1.293 to 0.005 lower)	⊕ ⊕ ⊕ ⊝ MODERATE ^a^

Exercise vs. control—outcome: pain (measured with: VAS, BPI, NPRS; better indicated by lower values)											
6 [29–34]	randomized trials	very serious^b^	very serious^c^	no serious indirectness	no serious imprecision	undetected	204	202	–	SMD's g 1.000 lower (1.481 to 0.519 lower)	⊕ ⊝ ⊝ ⊝ VERY LOW ^b, c^

Acupuncture vs. control—outcome: pain (measured with: NPRS, BPI-SF; better indicated by lower values)											
2 [25, 38]	randomized trials	very serious^d^	no serious inconsistency	no serious indirectness	no serious imprecision	strongly suspected^e^	24	31	–	SMD's g 0.817 lower (1.357 to 0.278 lower)	⊕ ⊝ ⊝ ⊝ VERY LOW ^d, e^

*CDT* Complex decongestive therapy, *NMT* Neuromuscular taping, *SMD* Standardized mean difference

^a^ Lack of allocation concealment in one trial [31, 35]. Lack of therapist blinding in two trials [31, 35, 36, 42]. Lack of assessor blinding in one trial [31, 35]. Analysis not performed on an intention-to-treat basis in one trial [31, 35]

^b^ Lack of allocation concealment in three trials [32–35, 38, 40]. Loss to follow-up in three trials [28–30, 33, 38]. Lack of therapist blinding in five trials [28–32, 34, 35, 40]. Lack of assessor blinding in two trials [32, 34, 35, 40]. Analysis not performed on an intention-to-treat basis in three trials [29, 30, 32, 33, 35, 38]

^c^ Statistical heterogeneity: high I^2^ (79.78%)

^d^ Lack of allocation concealment in two trials [25, 26, 38]. Loss to follow-up in one trial [26, 38]. Lack of therapist and assessor blinding in two trials [25, 26, 38]. Analysis not performed on an intention-to-treat basis in one trial [25]

^e^ Lack of disclosure of conflict of interest [25]

^f^ Lack of allocation concealment in two trials [34, 37, 41, 42]. Loss to follow-up in one trial [34, 41]. Lack of therapist blinding in two trials [34, 37, 41, 42]. Lack of assessor blinding in one trial [34, 41]. Analysis not performed on an intention-to-treat basis in two trials [34, 37, 41, 42]

^g^ Lack of therapist and assessor blinding[32, 40]

^h^ Lack of therapist blinding (Ligabue et al., 2019) [39]

^i^ Lack of allocation concealment in one trial [28, 41]. Loss to follow-up in three trials [21, 26, 27, 33, 36]. Lack of therapist blinding in four trials [21, 26–28, 33, 36, 41]. Lack of assessor blinding in one trial [28, 41]. Analysis not performed on an intention-to-treat basis in two trials [21, 26, 33]

^j^ Statistical heterogeneity: very high *I*^2^ (87.76%)

^k^ Statistical heterogeneity: substantial *I*^2^ (72.81%)

^l^ Statistical heterogeneity: high *I*^2^ (50.89%)

Given the serious risk of bias as revealed by the GRADE assessment and the PEDro scale, the overall GRADE quality of evidence for the included trials ranged from very low to moderate. Inconsistency, which was not assessed in the PEDro scale, could be another factor underlying the decline in GRADE quality. Very serious inconsistency was recognized in the pooled analyses from exercise trials for both QoL and pain measures due to the large variations in point estimates, wide CI and statistical heterogeneity across trials [22]. One trial was downgraded for publication bias because a conflict of interest was not reported [25]. Since the number of participants included in this meta-analytic review is more than the sample size necessary for an adequately powered trial, this OIS criterion was met and therefore trials were not downgraded for imprecision. Furthermore, none of the included trials were downgraded for indirectness.

### Effects of intervention on QoL

#### Exercise vs. control

Four trials [21, 26–28] compared the effect of exercise to a control condition on general, physical, and mental health components of QoL. The types of exercises included aerobic exercise, resistance training [21, 26–28], and aqua fitness exercise [26]. Aerobic exercise [28] was performed on the treadmill at an intensity of 60%–80% heart rate maximum. Resistance training [26] included exercises for the large muscles of the upper and lower limbs progressing from two sets of 12 repetitions at 50–60% one repetition maximum (RM) to three sets of 10 repetitions at 60–80% 1RM, over a period of 2 years. Aqua fitness exercise [26] consisted of aerobic and resistance exercise for the chest, shoulder, lower limbs, and core muscles. The exercise parameters in the four trials ranged from 30 to 60 min sessions, performed two to five times per week for a duration of 3 months to 2 years. Of the four trials, three [21, 27, 28] measured QoL using the SF-36 and one [26] measured QoL using the SF-12.

**Fig. 2:**
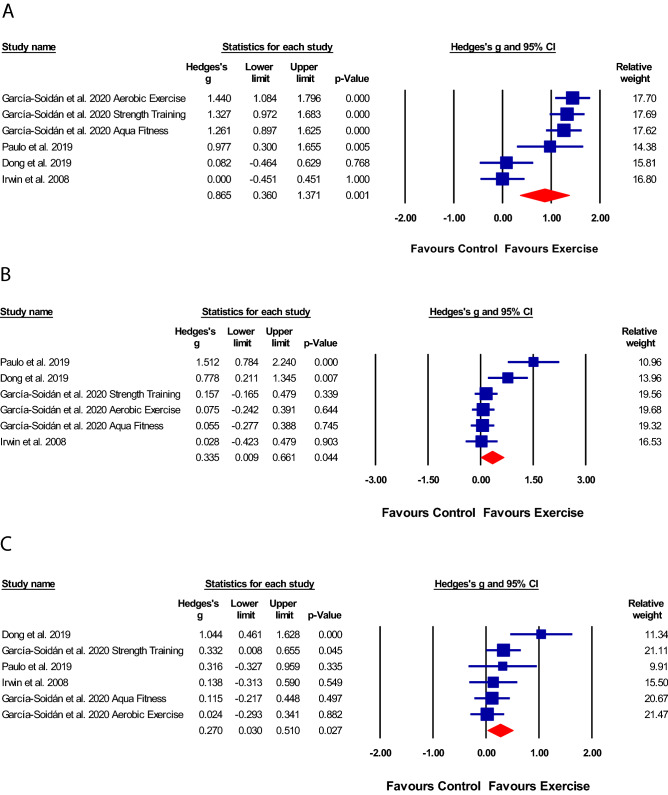
Effect of interventions on quality of life

The methodological quality of the four trials [21, 26–28] ranged from fair-to-high and the quality of the evidence was very low. The pooled analysis of the four trials (*n* = 451) revealed a statistically significant effect of the intervention on general [SMD 0.87 (95%CI: 0.36–1.37); *p* = 0.001; Fig. 2a], physical [SMD 0.34 (95%CI: 0.01–0.66); *p* = 0.044; Fig. 2b] and mental health components [SMD 0.27 (95%CI: 0.03–0.51); *p* = 0.027; Fig. 2c], when compared to the control condition.

### Effects of interventions on pain severity

#### Exercise vs. control

Six [29–34] trials evaluated the effectiveness of exercise on pain severity in women with PMPS. The types of exercise included in the six trials were aerobic exercise, resistance training [29, 32, 33] for the upper limbs (i.e., biceps, triceps, shoulder abductors and extensors, scapular muscles), lower limbs, and trunk muscles, hydrotherapy [31], stretching exercises, and nerve gliding exercise [30]. Of the six trials, one trial [33] measured pain severity with both VAS and BPI, two trials [29, 30] used NPRS, and three trials [31, 32, 34] utilized only VAS.

**Fig. 3:**
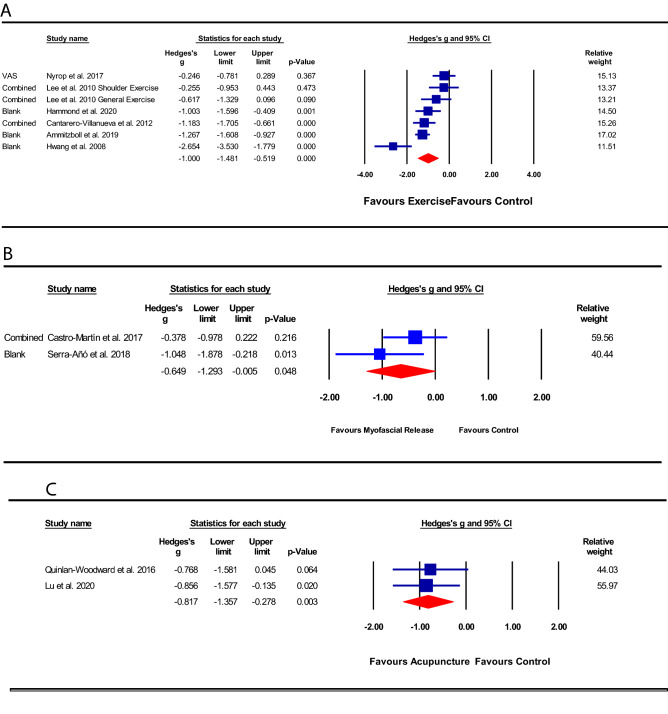

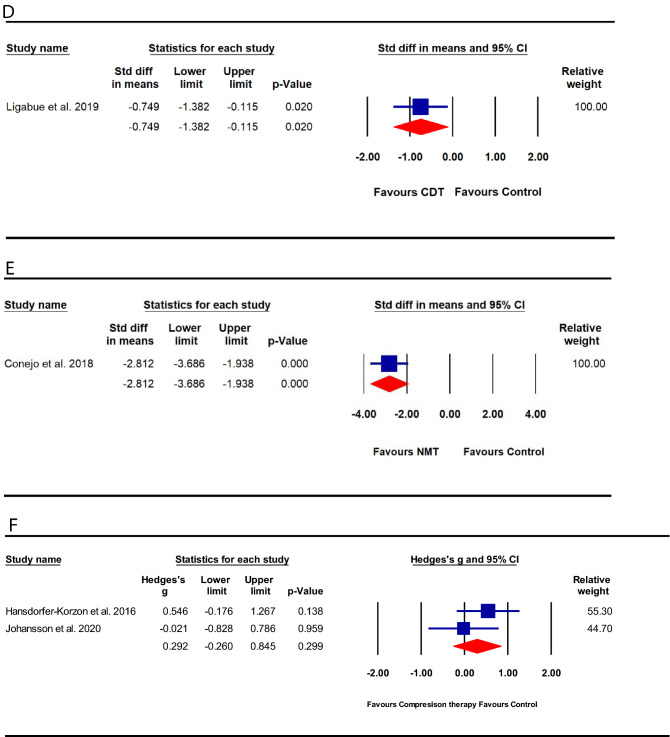
Effect of interventions on pain severity

The methodological quality of the six exercise trials [29–34] ranged from fair-to-high and the grade quality of the evidence was very low. Of these six trials, one trial [33] included three groups (scapula-oriented shoulder exercise, general exercise, and a control group), and data from the two exercise intervention groups versus the same control group were included individually. Pooled analysis of the six trials (*n* = 406) revealed a significantly greater reduction in pain severity in the intervention group than the control group [SMD − 1.00 (95%CI: − 1.48 to − 0.52); *p* < 0.001; Fig. 3a].

#### Myofascial release vs. placebo

Two [35, 36] of the 14 included trials compared the effectiveness of myofascial release to placebo. In both trials, the myofascial release therapy was applied using the Pilat approach [37] to the upper thoracic and upper limb region, for 30–40 min per session. The methodological quality of the two trials [35, 36] ranged from fair-to-high and the grade quality of evidence was moderate. Pooled analysis of the data from these two trials (*n* = 45) showed a statistically significant effect of the intervention compared to placebo [SMD − 0.65 (95%CI: − 1.29 to − 0.01); *p* = 0.04; Fig. 3b].

#### Acupuncture vs. control

Data were pooled from two trials [25, 38] of fair methodological quality and very low-grade evidence comparing the effect of acupuncture to usual care. One of the two trials provided electroacupuncture in 30-min sessions at an alternating frequency of 2–10 Hz, and participants received a total of 18 acupuncture treatments over the course of 8 weeks [38]. However, the other [25] trial provided inadequate intervention-related information such as the acupoints used, the depth of insertion, and whether needle stimulation was elicited. Pooled analysis (*n* = 55) revealed a statistically significant reduction in pain severity in the acupuncture group [SMD − 0.82 (95%CI: − 1.36 to − 0.29); *p* = 0.003; Fig. 3c] than in the control group.

#### Self-administered CDT vs. usual care

One small trial [39] (*n* = 41) of high methodological quality and moderate-grade evidence compared the effect of CDT to usual care; and utilized the NPRS to measure pain severity. The trial provided ten sessions of CDT (each lasting for 1.5 h) over the course of four weeks. The trial showed a statistically significant effect of CDT compared to usual care control [standard mean difference (SMD) − 0.75 (95%CI: − 1.38 to − 0.12]; *p* = 0.020; Fig. 3d].

#### NMT vs. sham-NMT

One small trial [40] (*n* = 40) of high methodological quality and moderate-grade evidence compared the effect of NMT to sham-NMT on pain severity. NMT was applied to the cervical, wrist forearm, and lumbosacral regions for three 7-day sessions over the course of 5 weeks. The trial showed a statistically significant effect of NMT compared to sham [SMD − 2.81 (95%CI: − 3.69 to − 1.94); *p* < 0.001; Fig. 3e].

#### Compression therapy vs. control

Data were pooled from two [41, 42] trials with low-grade evidence and a range of poor-to-high methodological quality comparing the effect of compression therapy to a control condition. One [41] trial provided a compression corset, while the other [42] provided participants with a compression-type sports bra. Both trials utilized the VAS for measuring pain severity. Pooled analysis (*n* = 60) revealed a non-significant effect of the intervention compared to control [SMD 0.29 (95%CI: − 0.26 to 0.85); *p* = 0.299; Fig. 3f].

## Discussion

This meta-analytic review evaluated the efficacy of physical therapy interventions compared to control for the management of upper quadrant pain in PMPS. Eighteen trials met the inclusion criteria and were included in the meta-analysis.

The pooled analysis of data from four trials [21, 26–28] of fair-to-high methodological quality and very-low-grade quality of evidence showed a significant effect of exercise compared with control conditions on the general, physical, and mental health components of QoL in women with PMPS. The results obtained in this meta-analytic review for QoL concur with the results of a previous meta-analytic review by Zeng et al. [9], who evaluated the efficacy of exercise interventions on QoL among breast cancer survivors. The effect size and the 95% CI were small in both reviews, indicating a clinically insignificant effect. Similar to the current paper, the previous review [9] also identified aerobic training combined with resistance training as being efficacious for improving the QoL in women with PMPS. However, the current review provides evidence for the efficacy of additional therapies, such as nerve gliding and water exercises, for alleviating PMPS, which were not evaluated in the previous review. Although the beneficial effects of exercise interventions on QoL were significant in this meta-analysis, definitive conclusions cannot be drawn due to the methodological flaws of the included trials, the failure of the 95% CI to exclude a clinically trivial effect, and the very low quality of evidence in the included studies. However, exercise is a low-risk intervention, and a recent systematic review [43] found that exercise was generally safe for women with breast cancer. Therefore, exercise interventions may be considered in clinical settings to improve QoL in women with PMPS.

Although exercise interventions showed positive effects on QoL, some demographic factors, such as age [44], race, and ethnicity [45], may also affect the evaluation of QoL in this population. Studies report that age may influence how breast cancer affects QoL in women; however, the reported effects of breast cancer on younger and older women have been contradictory. Some studies found that older women reported poorer QoL due to a sedentary lifestyle [44, 45], whereas other studies reported that women younger than 50 years report a greater QoL disturbance than those older than 50 years [46–49]. Younger women have been reported to lose more workdays and experience child-care problems that affect their QoL [46]. Breast cancer survivors from racially and ethnically diverse populations are associated with lower levels of physical activity and higher rates of obesity, which are commonly associated with poorer QoL [44, 45]. QoL also varies relative to socioeconomic status among breast cancer survivors [50], with low socioeconomic status associated with poorer QoL [44, 50]. Therefore, future RCTs must be carefully designed and include statistical methods to control for potentially confounding variables that may affect QoL measures in this population.

The use of generic measures of QoL in the current review does not allow for the determination of which disease-specific symptoms contribute the most to the limitations on physical functioning and psychological well-being among women with PMPS. The identification of disease-specific symptoms that impact the QoL among women with PMPS is important for treatment planning and goal setting in clinical practice. Future trials of physical therapy interventions for PMPS are recommended to utilize psychometrically valid and disease-specific outcome measures to evaluate QoL. The exercise parameters (frequency, intensity, duration, and time per session) in the exercise trials included in the current meta-analytic review varied greatly, minimizing the applicability of the findings to clinical settings. Future research remains necessary to determine the optimal exercise types and parameters that will improve QoL among women with PMPS.

The pooled analysis of data from six exercise trials [29–34] of fair-to-high methodological quality and very-low-grade quality of evidence showed a significant effect of exercise interventions on pain severity compared with control conditions for women with PMPS. These results concur with the results reported in the systematic review by Tatham et al. [51]. Tatham et al. [51] evaluated the efficacy of aerobic exercise and strength training on post-mastectomy shoulder pain but lacked a quantitative analysis. However, the current paper summarizes the evidence quantitatively and provides evidence for the efficacy of other therapies, such as nerve gliding and water exercises. Another contribution of the current meta-analytic review is the provision of new evidence regarding the reduction of upper quadrant pain in PMPS, whereas the previous review by Tatham et al. [51] only evaluated shoulder pain.

The effect size reported in the current meta-analytic review are below the clinically worthwhile threshold of 2 on a 0–10 VAS/NPRS scale [52], indicating a clinically insignificant effect. Therefore, further data in this area remain necessary to confirm the effectiveness of exercise on pain severity in women with PMPS. The exercise intervention parameters in the included trials of the current meta-analytic review varied greatly, which minimized the applicability of these findings to clinical settings. Future studies are required to determine the optimal types of exercise and exercise parameters for improving pain severity in women with PMPS.

The pooled analysis of data from two small myofascial release trials [35, 36] of fair-to-high methodological quality, moderate-grade and two small acupuncture trials [25, 38] of fair methodological quality, very-low-grade showed significantly increased reductions in pain severity in the intervention groups than in the control group. However, the mean estimate of the effect of myofascial release (0.65) and acupuncture interventions was (0.82) small. Due to the small effect size, the methodological quality of the included studies, and the sample size, we are unable to make any recommendations regarding the efficacy of myofascial release or acupuncture for the treatment of PMPS. Further research with larger sample sizes and carefully planned designs are required to confirm the effects of myofascial release and acupuncture for the treatment of PMPS.

This meta-analytic review has several strengths. This meta-analytic review identified significant benefits for several physical therapy interventions—including exercise therapy, myofascial release and acupuncture—in improving the QoL and reducing pain severity in women with PMPS. A comprehensive search strategy was utilized to identify trials evaluating the effectiveness of physical therapy interventions for the treatment of upper quadrant pain in women with PMPS. More than half of the studies included in the current meta-analytic review were of high methodological quality. Furthermore, more than half of the studies reported assessor blinding, thereby minimizing bias. The current meta-analytic review has some limitations: we could not evaluate the impact of publication bias due to the small number of studies included in each meta-analysis (a minimum of 8–10 studies are necessary to generate a funnel plot to assess publication bias). The second limitation is that the current meta-analytic review utilized generic outcome measures for QoL rather than disease-specific measures, which minimizes the applicability and generalizability of the findings to clinical settings and women with PMPS. Other limitations include the small sample sizes in some of the included trials, the small number of articles included in some meta-analyses, and the low methodological quality of some of the included studies. Therefore, adequately powered RCTs of high methodological quality remain necessary for future analysis to allow for the generation of appropriate future recommendations. These improvements could increase the specific understanding of the efficacy of physical therapy interventions in clinical practice.

## Conclusions

Meta-analysis revealed statistically significant effects of exercise compared to control in improving both overall QoL and pain. Exercise is a low-cost and safe intervention and could, therefore, be considered an essential component of QoL and pain management among women with PMPS. The exercise intervention parameters of the included trials in the current meta-analytic review varied greatly. Further research is required to determine the optimal parameters for exercise interventions designed to improve QoL and pain severity in women with PMPS. Our meta-analysis showed positive treatment effects for acupuncture and myofascial release for PMPS; however, due to the effect sizes, methodological qualities, grade of evidence, and sample sizes in the included trials, we are unable to make any recommendations regarding the efficacy of these interventions for the treatment of PMPS. Future research is required to investigate the effect of physical therapy interventions on QoL and surgery-related arm symptoms that contribute the most to the limitations on physical functioning and psychological well-being among women with PMPS.

## Supplementary Information

Below is the link to the electronic supplementary material.

Supplementary file1 (DOCX 48 kb)

## Abbreviations

BPI: Brief pain inventory
CDT: Complex decongestive therapy
GRADE: Grading of recommendations, assessment, development, and evaluation
NPRS: Numeric Pain Rating Scale
NMT: Neuromuscular taping
OIS: Optimal information size
PEDro: Physiotherapy evidence database
PMPS: Post-mastectomy pain syndrome
PRISMA: Preferred reporting items for systematic reviews and meta-analyses
QoL: Quality of life
RCT: Randomized controlled trial
RM: Repetition maximum
SD: Standard deviation
SF-12: 12-Item short form survey
SF-36: 36-Item short form survey
SMD: Standardized mean difference
VAS: Visual Analog Scale
