# Correction to: Efficacy of physical therapy interventions on quality of life and upper quadrant pain severity in women with post-mastectomy pain syndrome: a systematic review and meta-analysis

**DOI:** 10.1007/s11136-022-03104-3

**Published:** 2022-02-14

**Authors:** Priya Kannan, Hiu Ying Lam, Tsz Kiu Ma, Chiu Ngai Lo, Ting Yan Mui, Wing Yan Tang

**Affiliations:** Department of Rehabilitation Sciences, The Hong Kong Polytechnic University, Hung Hom, Kowloon, Hong Kong

## Correction to: Quality of Life Research https://doi.org/10.1007/s11136-021-02926-x

The article “Efficacy of physical therapy interventions on quality of life and upper quadrant pain severity in women with post‑mastectomy pain syndrome: a systematic review and meta‑analysis”, written by Priya Kannan, Hiu Ying Lam, Tsz Kiu Ma, Chiu Ngai Lo, Ting Yan Mui and Wing Yan Tang, was originally published electronically on the publisher’s internet portal on 29 June 2021 without open access. With the author(s)’ decision to opt for Open Choice the copyright of the article changed on 28 January 2022 to © The Author(s) 2022 and the article is forthwith distributed under a Creative Commons Attribution 4.0 International License, which permits use, sharing, adaptation, distribution and reproduction in any medium or format, as long as you give appropriate credit to the original author(s) and the source, provide a link to the Creative Commons licence, and indicate if changes were made. The images or other third party material in this article are included in the article’s Creative Commons licence, unless indicated otherwise in a credit line to the material. If material is not included in the article’s Creative Commons licence and your intended use is not permitted by statutory regulation or exceeds the permitted use, you will need to obtain permission directly from the copyright holder. To view a copy of this licence, visit http://creativecommons.org/licenses/by/4.0.

The original article has been corrected.

