# Supplementary material for: Efficacy of physical therapy interventions on quality of life and upper quadrant pain severity in women with post-mastectomy pain syndrome: a systematic review and meta-analysis
Source: Qual Life Res. 2021 Jun 29;31(4):951–73. doi: 10.1007/s11136-021-02926-x (PMC8960660; doi:10.1007/s11136-021-02926-x)
Supplement: Supplementary file 1 — Supplementary file1 (DOCX 48 kb) [file 11136_2021_2926_MOESM1_ESM.docx]

**Supplementary data**

**Supplementary Appendix 1.** Search term and search strategy for Medline

| **Subject areas** | **Search terms used** | **Limits** |
| --- | --- | --- |
| Breast cancer (AND) | quality of life (OR) Chronic postmastectomy pain (OR) postmastectomy pain syndrome (OR) breast cancer surgery (OR) ( mastectomy or breast surgery or breast removal ) (OR) postmastectomy pain (OR) breast surgery (OR) post-mastectomy pain (OR) breast cancer | - Human studies - Full-text RCTs conducted on females - English or Chinese language studies published in peer-reviewed journals - Date of publication: 1975 (database inception) to 2020 |
| Physical therapy interventions (AND) | Myofascial massage (OR) Pulsed high-intensity laser therapy (OR) low level laser therapy (OR) (exercise or physical activity) (OR) resistive training (OR) (aerobic exercise or aerobic training or physical activity or exercise or physical exercise) (OR) (manual therapy or mobilization or manipulation) (OR) (compression therapy, or compression garments, or bandaging) (OR) (acupuncture or acupuncture therapy or acupuncture treatment) (OR) (kinesio taping or kinesiotape) (OR) (transcutaneous electrical nerve stimulation or tens |  |
| Randomized controlled trial | randomized controlled study (OR) Randomized controlled clinical trial (OR) (randomized controlled trials or rct or randomised control trials) (OR) randomized trial (OR) Randomized sham-controlled trial (OR) random allocation |  |

Note: RCTs: Randomized Controlled Trials.

**Supplementary Appendix 2.** Excluded studies and reasons for exclusion in full-text screening

| **No.** | **First author and year** | **Study title** | **Reasons for exclusion** |
| --- | --- | --- | --- |
|  | Oh 2011 | Acupuncture research protocol: Feasibility of use of acupuncture for treatment of arthralgia secondary to aromatase inhibitor therapy in women with early breast cancers | Study protocol |
|  | Lauridsen 2000 | Physiotherapy treatment of late symptoms following surgical treatment of breast cancer | Two active treatments are compared |
|  | Kozanoglu 2009 | Efficacy of pneumatic compression and low-level laser therapy in the treatment of postmastectomy lymphedema: a randomized controlled trial |  |
|  | Jprn 2016 | Effect of education, and exercise program and on long term physical activity in post-operative breast cancer patients: randomised controlled trial | Unfinished trial |
|  | Nct 2019 | Effects of Exercise in Patients With Metastatic Breast Cancer |  |
|  | Anderson 2012 | A randomized trial of exercise on well-being and function following breast cancer surgery: The RESTORE trial | Ineligible results for data extraction |
|  | Ibrahim 2018 | The long-term effects of posttreatment exercise on pain in young women with breast cancer |  |
|  | Kaviani 2006 | Low-level laser therapy in management of postmastectomy lymphedema |  |
|  | Arinaga 2019 | The 10-Min Holistic Self-Care for Patients with Breast Cancer-Related Lymphedema: pilot Randomized Controlled Study | Not PT intervention |
|  | Johansson 2020 | Compression Treatment of Breast Edema: A Randomized Controlled Pilot Study |  |
|  | Basen-Engquist 2006 | Randomized pilot test of a lifestyle physical activity intervention for breast cancer survivors |  |
|  | Rosner 2011 | Evaluation of a Nordic Walking Program on Shoulder Joint Mobility and Isometric Force in Breast Cancer Patients | Non-English study |
|  | Rzepka 2010 | Fatigue among breast cancer patients |  |
|  | de León Carrillo 2016 | A randomised controlled trial of the efficacy of a controlled compression garment versus compressive bandaging in the immediate postoperative period after breast cancer-conserving surgery |  |
|  | ChiCtr 2019 | The effectiveness of dancing program on relieving the symptom cluster of fatigue-sleep disturbance-depression among breast cancer patients undergoing adjuvant chemotherapy: a randomized controlled trial | No full-text |
|  | / | Electrical stimulation effect to diminish pain on arm in women undergoing surgery for breast cancer |  |
|  | Gibbs 2011 | High vs low intensity resistance exercise in late stage breast cancer patients with lymphedema: a randomised controlled trial |  |
|  | Kwiatkowski 2013 | Long term improved quality of life by a 2-week group physical and educational intervention shortly after breast cancer chemotherapy completion. Results of the 'Programme of Accompanying women after breast Cancer treatment completion in Thermal resorts' (PACThe) randomised clinical trial of 251 patients |  |
|  | Kirkham 2019 | Maintenance of fitness and quality-of-life benefits from supervised exercise offered as supportive care for breast cancer |  |
|  | / | Acupuncture for Peripheral Neuropathy Induced by Paclitaxel in Early Stage Breast Cancer |  |
|  | Mazuquin 2020 | PRevention of shoulder ProblEms TRial (PROSPER): exercise to prevent shoulder problems in patients undergoing breast cancer treatment |  |
|  | King 2012 | Compression garments versus compression bandaging in decongestive lymphatic therapy for breast cancer-related lymphedema: a randomized controlled trial | Combination of treatments for the intervention group |
|  | Tantawy 2016 | The effect of Kinesio Taping with exercise compared with exercise alone on pain, range of motion, and disability of the shoulder in postmastectomy females: a randomized control trial |  |
|  | Ho 2016 | Effects of a Short-Term Dance Movement Therapy Program on Symptoms and Stress in Patients with Breast Cancer Undergoing Radiotherapy: A Randomized, Controlled, Single-Blind Trial |  |
|  | Beurskens 2007 | The efficacy of physiotherapy upon shoulder function following axillary dissection in breast cancer, a randomized controlled study |  |
|  | Mock 2005 | Exercise manages fatigue during breast cancer treatment: A randomized controlled trial |  |
|  | Buffart 2012 | How does exercise improve cancer survivors, quality of life? |  |
|  | Ebid 2015 | Long-term effect of pulsed high-intensity laser therapy in the treatment of post-mastectomy pain syndrome: a double blind, placebo-control, randomized study |  |
|  | Baxter 2018 | Low level laser therapy for the management of breast cancer-related lymphedema: A randomized controlled feasibility study |  |
|  | Ezzo 2015 | Manual lymphatic drainage for lymphedema following breast cancer treatment |  |
|  | Martín 2011 | Manual lymphatic drainage therapy in patients with breast cancer related lymphoedema |  |
|  | De Groef 2017 | Myofascial techniques have no additional beneficial effects to a standard physical therapy programme for upper limb pain after breast cancer surgery: a randomized controlled trial |  |
|  | Demark-Wahnefried 2015 | Quality of life outcomes from the Exercise and Nutrition Enhance Recovery and Good Health for You (ENERGY)-randomized weight loss trial among breast cancer survivors |  |
|  | Testa 2014 | Strengths of early physical rehabilitation programs in surgical breast cancer patients: results of a randomized controlled study |  |
|  | De Groef 2018 | Effect of myofascial techniques for treatment of persistent arm pain after breast cancer treatment: randomized controlled trial |  |
|  | Paice 2011 | Chronic treatment-related pain in cancer survivors | Non-RCT |
|  | Ye 2015 | Clinical study of acupuncture intervention in muscle, bone and joint pain caused by aromatase inhibitors in the treatment of breast cancer |  |
|  | Tunay 2012 | The Effect of Multidimensional Physiotherapy Program on Shoulder Function, Pain, and Lymphedema After Surgery in Elderly Breast Cancer Patients |  |
|  | O'Neill 2006 | The Effects of Complete Decongestive Physical Therapy Treatment On Edema Reduction, Quality of Life, and Functional Ability of Persons with Upper Extremity Lymphedema |  |
|  | Sung 2015 | Factors associated with persistent pain following breast cancer treatment |  |
|  | Hardy 2006 | Multiple benefits of exercise for breast cancer survivors |  |
|  | Mock 1994 | A nursing rehabilitation program for women with breast cancer receiving adjuvant chemotherapy |  |
|  | Kwekkeboom 2001 | Pain Management Strategies Used by Patients With Breast and Gynecologic Cancer With Postoperative Pain |  |
|  | Le Vu 1997 | Physiotherapy after surgery for breast cancer |  |
|  | Cave 2006 | Physiotherapy improves shoulder function after treatment in women with early breast cancer |  |
|  | Robertson 1999 | Pneumatic compression therapy does not reduce chronic post-mastectomy lymphoedema |  |
|  | Noble 2012 | UW WELL-FIT- the impact of supervised exercise programs on physical capacity and quality of life in individuals receiving treatment for cancer |  |
|  | Moseley 2005 | The effect of gentle arm exercise and deep breathing on secondary arm lymphedema |  |
|  | Sander 2008 | A safe and effective upper extremity resistive exercise program for woman post breast cancer treatment |  |
|  | Kalda 1999 | The effect of upper body exercise on secondary lymphedema following breast cancer treatment |  |
|  | Kolden 2002 | A pilot study of group exercise training (GET) for women with primary breast cancer: feasibility and health benefits |  |
|  | Spector 2014 | A pilot study of a home-based motivational exercise program for African American breast cancer survivors: clinical and quality-of-life outcomes |  |
|  | Cuesta-Vargas 2014 | A multimodal physiotherapy programme plus deep water running for improving cancer-related fatigue and quality of life in breast cancer survivors |  |
|  | Gautam 2011 | Effect of home-based exercise program on lymphedema and quality of life in female postmastectomy patients: Pre-post intervention study |  |
|  | Mondry 2004 | Prospective trial of complete decongestive therapy for upper extremity lymphedema after breast cancer therapy |  |
|  | Mallory 2015 | Acupuncture in the postoperative setting for breast cancer patients: A feasibility study |  |
|  | Cornette 2013 | Adapted physical activity effect on aerobic function in patients with breast cancer treated with adjuvant or neoadjuvant chemotherapy | Conference abstract/poster |
|  | Irwin 2012 | Aromatase inhibitors, arthralgias, and exercise in breast cancer survivors |  |
|  | Ozsoy 2016 | Comparison of kinesiotaping with compression garment in the treatment of lymphedema after breast cancer: a preliminary report |  |
|  | Choo 2018 | Early impact of a 12-week exercise intervention program on mental health, quality of life and immune markers in early stage breast cancer patients |  |
|  | Haas 2019 | Effect of exercise during adjuvant chemotherapy for breast cancer |  |
|  | Waart 2014 | Effect of low versus high intensity physical exercise during chemotherapy on physical fitness, fatigue and chemotherapy completion rates: results of a randomized, controlled trial |  |
|  | De Groef 2016 | Effect of myofascial techniques in addition to standard physical therapy for treatment of pain and upper limb problems in breast cancer survivors: Randomized controlled trial |  |
|  | Zissiadis 2015 | Efficacy and feasibility of a home based exercise program for reducing cancer related fatigue in breast cancer patients undergoing curative radiotherapy: a randomised controlled trial |  |
|  | Cormie 2014 | Efficacy of home based exercise for reducing cancer related fatigue in breast cancer patients undergoing radical radiotherapy: A randomised controlled trial |  |
|  | Hayes 2012 | Evaluating telephone versus face-to-face modes of exercise intervention delivery to women during and following treatment for breast cancer |  |
|  | Sheehan 2016 | Evaluation of a Sustainable Intervention using Exercise-for Cancer Fatigue (ESIE-CF Trial) |  |
|  | Adamsen 2019 | Exercise in cancer survivors center for integrated rehabilitation of cancer patients (CIRE) illuminates the role that exercise and supportive care can play for cancer survivors |  |
|  | Casla 2015 | Exercise intervention to run away from breast cancer treatment side effects: an integrative approach |  |
|  | Lacomba 2012 | Myofascial pain syndrome in breast cancer survivors: Double-blind, randomized, clinical trial of the efficacy of physical therapy |  |
|  | Leibbrand 2010 | Nordic Walking - Trend or ideal performance training in breast cancer? |  |
|  | Petrella 2012 | A pilot study evaluating the benefits and feasibility of an exercise program for breast cancer patients receiving adjuvant chemotherapy |  |
|  | Bruce 2019 | Postoperative pain after breast cancer surgery: the UK Prevention of Shoulder Problems Trial (UK PROSPER) |  |
|  | Bruce 2020 | Prevention of shoulder problems trial (UK-PROSPER): Exercise to prevent shoulder problems in patients undergoing breast cancer treatment |  |
|  | Hershman 2018 | Randomized blinded sham- and waitlist-controlled trial of acupuncture for joint symptoms related to aromatase inhibitors in women with early stage breast cancer (S1200) |  |
|  | Menzel 2013 | Randomized controlled clinical study of exercise program on functional and emotional response in women with surgery breast cancer |  |
|  | Frank 2017 | A randomized controlled trial comparing acupuncture versus usual care for the treatment of aromatase inhibitor-induced arthralgia (AIIA) in women with early-stage breast cancer |  |
|  | Beith 2011 | A randomized trial assessing the use of electro-acupuncture for aromatase inhibitor-induced arthralgia |  |
|  | Tajaesu 2017 | Randomized trial of exercise intervention vs. usual care for breast cancer patients with aromatase inhibitor to prevent and improve the aromatase inhibitor induced arthralgia |  |
|  | Lohrisch 2011 | Randomized Trial of Exercise TRandomized trial of exercise versus control for musculoskeletal symptoms from adjuvant anastrozole (A) for postmenopausal early breast cancer (PEBC) |  |
|  | Irwin 2013 | Randomized trial of exercise vs. usual care on aromatase inhibitor-associated arthralgias in women with breast cancer: The hormones and physical exercise (HOPE) study |  |
|  | Palesh 2015 | RCT utilizing acupuncture for management of insomnia associated with cancer |  |
|  | Espejo-Reina 2018 | SINCROBOX® as a method to improve shoulder’s function in mastectomized women |  |
|  | Kipling 2015 | Supervised exercise for older women treated for breast cancer. Preliminary results from a pilot randomised controlled trial |  |
|  | Hansdorfer-Korzon 2018 | Usefulness of low compression corsets in prevention of lym-phoedema in patients after axillary lymphadenectomy |  |
|  | Johns 2020 | Acceptance and commitment therapy for breast cancer survivors with fear of cancer recurrence: A 3-arm pilot randomized controlled trial | Ineligible participants^*^ |
|  | Pantuso 2018 | Acupuncture for aromatase inhibitor associated pain in breast cancer patients |  |
|  | Oh 2013 | Acupuncture for treatment of arthralgia secondary to aromatase inhibitor therapy in women with early breast cancer: Pilot study |  |
|  | Dieli-Conwright 2018 | Aerobic and resistance exercise improves physical fitness, bone health, and quality of life in overweight and obese breast cancer survivors: A randomized controlled trial 11 Medical and Health Sciences 1117 Public Health and Health Services |  |
|  | Alimi 2003 | Analgesic Effect of Auricular Acupuncture for Cancer Pain: A Randomized, Blinded, Controlled Trial |  |
|  | Fillion 2008 | A brief intervention for fatigue management in breast cancer survivors |  |
|  | Litterini 2008 | The change in fatigue, strength, and quality of life following a physical therapist prescribed exercise program for cancer survivors |  |
|  | Kenzik 2018 | Changes in Body Mass Index and Physical Activity Predict Changes in Vitality During a Weight Loss Trial in Breast Cancer Survivors |  |
|  | Badger 2007 | Depression and anxiety in women with breast cancer and their partners |  |
|  | Bao 2013 | A dual-center randomized controlled double blind trial assessing the effect of acupuncture in reducing musculoskeletal symptoms in breast cancer patients taking aromatase inhibitors |  |
|  | Frensham 2018 | Effect of a 12-Week Online Walking Intervention on Health and Quality of Life in Cancer Survivors: A Quasi-Randomized Controlled Trial |  |
|  | Reis 2018 | Effect of exercise on pain and functional capacity in breast cancer patients |  |
|  | McKenzie 2003 | Effect of upper extremity exercise on secondary lymphedema in breast cancer patients: a pilot study |  |
|  | Travier 2015 | Effects of an 18-week exercise programme started early during breast cancer treatment: a randomised controlled trial |  |
|  | Baruth 2015 | Effects of Home-Based Walking on Quality of Life and Fatigue Outcomes in Early Stage Breast Cancer Survivors: A 12-Week Pilot Study |  |
|  | Jacobsen 2013 | Effects of self-directed stress management training and home-based exercise on quality of life in cancer patients receiving chemotherapy: a randomized controlled trial |  |
|  | Duijts 2012 | Efficacy of cognitive behavioral therapy and physical exercise in alleviating treatment-induced menopausal symptoms in patients with breast cancer: Results of a randomized, controlled, multicenter trial |  |
|  | Sima 2009 | Efficacy of electroacupuncture for bone metastatic cancer patients with neuropathic pain: A randomized controlled trial |  |
|  | Midtgaard 2013 | Efficacy of multimodal exercise-based rehabilitation on physical activity, cardiorespiratory fitness, and patient-reported outcomes in cancer survivors: a randomized, controlled trial |  |
|  | Baglia 2019 | Endocrine-related quality of life in a randomized trial of exercise on aromatase inhibitor–induced arthralgias in breast cancer survivors |  |
|  | de Almeida 2018 | Evaluation of the Impact of Physical Exercise in Reducing Pain in Women Undergoing Mammography: A Randomized Clinical Trial |  |
|  | Witlox 2018 | Four-year effects of exercise on fatigue and physical activity in patients with cancer |  |
|  | Meraviglia 2013 | Health Promotion for Cancer Survivors: Adaptation and Implementation of an Intervention |  |
|  | Griffith 2009 | Impact of a walking intervention on cardiorespiratory fitness, self-reported physical function, and pain in patients undergoing treatment for solid tumors |  |
|  | Schuler 2017 | Impact of Different Exercise Programs on Severe Fatigue in Patients Undergoing Anticancer Treatment-A Randomized Controlled Trial |  |
|  | Godoy-Izquierdo 2017 | Improvements in health-related quality of life, cardio-metabolic health, and fitness in postmenopausal women after a supervised, multicomponent, adapted exercise program in a suited health promotion intervention: a multigroup study |  |
|  | Cormie 2013 | Is it safe and efficacious for women with lymphedema secondary to breast cancer to lift heavy weights during exercise: a randomised controlled trial |  |
|  | Kwiatkowski 2017 | Long-term improvement of breast cancer survivors' quality of life by a 2-week group physical and educational intervention: 5-year update of the 'PACThe' trial |  |
|  | Courneya 2008 | Moderators of the effects of exercise training in breast cancer patients receiving chemotherapy: A randomized controlled trial |  |
|  | Bao 2011 | A multi-center randomized controlled double blind trial assessing the effect of acupuncture in reducing musculoskeletal symptoms in breast cancer patients taking aromatase inhibitors: First interim analysis |  |
|  | Ridner 2013 | A pilot randomized trial evaluating low-level laser therapy as an alternative treatment to manual lymphatic drainage for breast cancer-related lymphedema |  |
|  | Crew 2007 | Pilot study of acupuncture for the treatment of joint symptoms related to adjuvant aromatase inhibitor therapy in postmenopausal breast cancer patients |  |
|  | Wilburn 2006 | A pilot, prospective evaluation of a novel alternative for maintenance therapy of breast cancer-associated lymphedema |  |
|  | Mao 2014 | A randomised trial of electro-acupuncture for arthralgia related to aromatase inhibitor use |  |
|  | Nyrop 2017 | Randomized Controlled Trial of a Home-Based Walking Program to Reduce Moderate to Severe Aromatase Inhibitor-Associated Arthralgia in Breast Cancer Survivors |  |
|  | McClure 2010 | Randomized controlled trial of the Breast Cancer Recovery Program for women with breast cancer-related lymphedema |  |
|  | Courneya 2009 | Randomized Controlled Trial of the Effects of Aerobic Exercise on Physical Functioning and Quality of Life in Lymphoma Patients |  |
|  | Irwin 2015 | Randomized Exercise Trial of Aromatase Inhibitor-Induced Arthralgia in Breast Cancer Survivors |  |
|  | Bernhorster 2011 | Randomized, Blinded, Sham-Controlled Trial of Acupuncture for the Management of Aromatase Inhibitor–Associated Joint Symptoms in Women With Early-Stage Breast Cancer |  |
|  | Serra 2018 | Resistance training reduces inflammation and fatigue and improves physical function in older breast cancer survivors |  |
|  | Jones 2014 | Safety and Efficacy of Aerobic Training in Patients With Cancer Who Have Heart Failure: An Analysis of the HF-ACTION Randomized Trial |  |
|  | Winkels 2017 | The women in steady exercise research (WISER) survivor trial: The innovative transdisciplinary design of a randomized controlled trial of exercise and weight-loss interventions among breast cancer survivors with lymphedema |  |
|  | Rostock 2013 | Chemotherapy-induced peripheral neuropathy in cancer patients: a four-arm randomized trial on the effectiveness of electroacupuncture |  |
|  | Oh 2013 | Acupuncture for treatment of arthralgia secondary to aromatase inhibitor therapy in women with early breast cancer: Pilot study |  |
|  | Battaglini 2006 | The effects of resistance training on muscular strength and fatigue levels in breast cancer patients |  |
|  | Reed 2020 | Group versus Individual Acupuncture (AP) for Cancer Pain: A Randomized Noninferiority Trial |  |
|  | Dong 2020 | A Longitudinal Study of a Multicomponent Exercise Intervention with Remote Guidance among Breast Cancer Patients |  |
|  | Xie 2010 | 169 patients with postoperative breast cancer on exercising the function of limbs and investigating quality of life: a clinical study | Ineligible outcome measures^#^ |
|  | Diaz 2017 | Accessory Joint and Neural Mobilizations for Shoulder Range of Motion Restriction After Breast Cancer Surgery: A Pilot Randomized Clinical Trial |  |
|  | Bloom 2013 | Bone health for younger women with breast cancer: effects of a YMCA-based intervention on exercise, bone density, body mass, and physical and mental health in a multi-ethnic sample |  |
|  | Tantawy 2019 | Comparative Study Between the Effects of Kinesio Taping and Pressure Garment on Secondary Upper Extremity Lymphedema and Quality of Life Following Mastectomy: a Randomized Controlled Trial |  |
|  | van Waart 2015 | Effect of Low-Intensity Physical Activity and Moderate- to High-Intensity Physical Exercise During Adjuvant Chemotherapy on Physical Fitness, Fatigue, and Chemotherapy Completion Rates: Results of the PACES Randomized Clinical Trial |  |
|  | Gol 2020 | Effect of massage therapy with and without elastic bandaging on pain, edema, and shoulder dysfunction after modified radical mastectomy: A clinical trial |  |
|  | Chen 2009 | The Effect of Regular Exercise on Quality of Life Among Breast Cancer Survivors |  |
|  | Wang 2011 | Effects of a 6-week walking program on Taiwanese women newly diagnosed with early-stage breast cancer |  |
|  | Yang 2011 | Effects of a home-based walking program on perceived symptom and mood status in postoperative breast cancer women receiving adjuvant chemotherapy |  |
|  | Nemli 2019 | Effects of exercise training and follow‐up calls at home on physical activity and quality of life after a mastectomy |  |
|  | Cornette 2016 | Effects of home-based exercise training on VO2 in breast cancer patients under adjuvant or neoadjuvant chemotherapy (SAPA): A randomized controlled trial |  |
|  | Zimmermann 2012 | Efficacy of manual lymphatic drainage in preventing secondary lymphedema after breast cancer surgery |  |
|  | Hayes 2013 | Exercise for health: a randomized, controlled trial evaluating the impact of a pragmatic, translational exercise intervention on the quality of life, function and treatment-related side effects following breast cancer |  |
|  | Crank 2007 | Exercise therapy in women treated for breast cancer |  |
|  | Daley 2004 | Exercise therapy in women who have had breast cancer: design of the Sheffield women's exercise and well-being project |  |
|  | Dash 2016 | An exercise trial to reduce cancer related fatigue in African American breast cancer patients undergoing radiation therapy: design, rationale, and methods |  |
|  | Mock 2001 | Fatigue and quality of life outcomes of exercise during cancer treatment |  |
|  | Mulero Portela 2008 | Feasibility of an exercise program for Puerto Rican women who are breast cancer survivors |  |
|  | Smith 2014 | A feasibility study to examine the role of acupuncture to reduce symptoms of lymphoedema after breast cancer: A randomised controlled trial |  |
|  | Campbell 2012 | Five year follow up of an exercise intervention during breast cancer treatment |  |
|  | Mutrie 2012 | Five-year follow-up of participants in a randomised controlled trial showing benefits from exercise for breast cancer survivors during adjuvant treatment. Are there lasting effects? |  |
|  | Bloomquist 2018 | Heavy-load lifting: Acute response in breast cancer survivors at risk for lymphedema |  |
|  | Mijwel 2019 | High-intensity exercise during chemotherapy induces beneficial effects 12 months into breast cancer survivorship |  |
|  | Mijwel 2018 | Highly favorable physiological responses to concurrent resistance and high-intensity interval training during chemotherapy: the OptiTrain breast cancer trial |  |
|  | Wenzel 2013 | Impact of a home-based walking intervention on outcomes of sleep quality, emotional distress, and fatigue in patients undergoing treatment for solid tumors |  |
|  | Briskin 2015 | Improvement of upper limb’s condition of Women with post mastectomy syndrome with the help of problem-oriented program of physical rehabilitation |  |
|  | Landry 2018 | Influence of an Adapted Physical Activity Program on Self-Esteem and Quality of Life of Breast Cancer Patients after Mastectomy |  |
|  | Schmidt 2017 | Influence of arm crank ergometry on development of lymphoedema in breast cancer patients after axillary dissection: A Randomized Controlled Trial |  |
|  | Haines 2010 | Multimodal exercise improves quality of life of women being treated for breast cancer, but at what cost? Randomized trial with economic evaluation |  |
|  | Massingill 2018 | Myofascial Massage for Chronic Pain and Decreased Upper Extremity Mobility After Breast Cancer Surgery |  |
|  | Marshall-McKenna 2014 | Myofascial release for women undergoing radiotherapy for breast cancer: A pilot study |  |
|  | Ochalek 2018 | Physical Activity With and Without Arm Sleeves: compliance and Quality of Life After Breast Cancer Surgery-A Randomized Controlled Trial |  |
|  | Yeh 2016 | Pilot Randomized Controlled Trial of Auricular Point Acupressure to Manage Symptom Clusters of Pain, Fatigue, and Disturbed Sleep in Breast Cancer Patients |  |
|  | Campbell 2005 | A pilot study of a supervised group exercise programme as a rehabilitation treatment for women with breast cancer receiving adjuvant treatment |  |
|  | Kim 2020 | Pre-post analysis of a social capital-based exercise adherence intervention for breast cancer survivors with moderate fatigue: a randomized controlled trial |  |
|  | Izgu 2019 | Prevention of chemotherapy-induced peripheral neuropathy with classical massage in breast cancer patients receiving paclitaxel: An assessor-blinded randomized controlled trial |  |
|  | Ammitzboll 2019 | Progressive resistance training to prevent arm lymphedema in the first year after breast cancer surgery: results of a randomized controlled trial |  |
|  | Rethorst 2018 | The Promoting Activity in Cancer Survivors (PACES) trial: a multiphase optimization of strategy approach to increasing physical activity in breast cancer survivors |  |
|  | Pinto 2003 | Psychological and fitness changes associated with exercise participation among women with breast cancer |  |
|  | Nedstrand 2006 | Psychological well-being improves in women with breast cancer after treatment with applied relaxation or electro-acupuncture for vasomotor symptom |  |
|  | Heim 2007 | Randomized controlled trial of a structured training program in breast cancer patients with tumor-related chronic Fatigue |  |
|  | Courneya 2003 | Randomized controlled trial of exercise training in postmenopausal breast cancer survivors: cardiopulmonary and quality of life outcomes |  |
|  | Ahmed 2006 | Randomized Controlled Trial of Weight Training and Lymphedema in Breast Cancer Survivors |  |
|  | Potthoff 2013 | Randomized controlled trial to evaluate the effects of progressive resistance training compared to progressive muscle relaxation in breast cancer patients undergoing adjuvant radiotherapy: the BEST study |  |
|  | Rabin 2016 | Randomized Trial of a Physical Activity and Meditation Intervention for Young Adult Cancer Survivors |  |
|  | Daley 2007 | Randomized Trial of Exercise Therapy in Women Treated for Breast Cancer |  |
|  | Rogers 2009 | A Randomized Trial to Increase Physical Activity in Breast Cancer Survivors |  |
|  | Molassiotis 2013 | A randomized, controlled trial of acupuncture self-needling as maintenance therapy for cancer-related fatigue after therapist-delivered acupuncture |  |
|  | Steindorf 2014 | Randomized, controlled trial of resistance training in breast cancer patients receiving adjuvant radiotherapy: results on cancer-related fatigue and quality of life |  |
|  | Hagstrom 2016 | Resistance training improves fatigue and quality of life in previously sedentary breast cancer survivors: a randomised controlled trial |  |
|  | Dini 1998 | The role of pneumatic compression in the treatment of postmastectomy lymphedema. A randomized phase III study |  |
|  | Box 2002 | Shoulder movement after breast cancer surgery: results of a randomised controlled study of postoperative physiotherapy |  |
|  | Rabelo Mendonça 2017 | TENS effects on dysesthesia and quality of life after breast cancer surgery with axilectomy: randomized controlled trial |  |
|  | Sprod 2010 | Three versus six months of exercise training in breast cancer survivors |  |
|  | de Rezende 2006 | Two exercise schemes in postoperative breast cancer: comparison of effects on shoulder movement and lymphatic disturbance |  |
|  | Bolam 2019 | Two-year follow-up of the OptiTrain randomised controlled exercise trial |  |
|  | Schmitz 2009 | Weight Lifting in Women with Breast- Cancer–Related Lymphedema |  |
|  | Crawford-Faucher 2011 | Women at risk of breast cancer-associated Lymphedema can safely lift weights |  |
|  | Swedborg 1993 | Lymphoedema post-mastectomy: is elevation alone an effective treatment? |  |
|  | Lee 2007 | Pectoral stretching program for women undergoing radiotherapy for breast cancer |  |
|  | Naumann 2012 | An individual-based versus group-based exercise and counselling intervention for improving quality of life in breast cancer survivors. A feasibility and efficacy study. |  |
|  | Ting 2017 | Acupuncture for breast cancer related lymphedema: a randomized controlled trial |  |
|  | Balzarini 1993 | Ultrasound therapy of chronic arm lymphedema after surgical treatment of breast cancer |  |
|  | Zhang 2020 | Electroacupuncture trigeminal nerve stimulation plus body acupuncture for chemotherapy-induced cognitive impairment in breast cancer patients: an assessor-participant blinded, randomized controlled trial |  |
|  | Jarvandi 2020 | Improving Lifestyle Behaviors After Breast Cancer Treatment Among African American Women With and Without Diabetes: Role of Health Care Professionals |  |

*Note*: *Ineligible participants: Women without upper quadrant pain; #Ineligible outcome measures: outcome measures outside of the scope of interest of the current meta-analytic review.
